# Recent Progress in Synthesis of Glycerol Carbonate and Evaluation of Its Plasticizing Properties

**DOI:** 10.3389/fchem.2019.00308

**Published:** 2019-05-24

**Authors:** Pascale de Caro, Matthieu Bandres, Martine Urrutigoïty, Christine Cecutti, Sophie Thiebaud-Roux

**Affiliations:** ^1^Laboratoire de Chimie Agro-industrielle, LCA, Université de Toulouse, INRA, Toulouse, France; ^2^Laboratoire de Chimie de Coordination, LCC, Université de Toulouse, CNRS, Toulouse, France

**Keywords:** glycerol, glycerol carbonate, transcarbonatation, plasticizer, green chemistry

## Abstract

The state of the art on the glycerol carbonate (GC) synthesis has been updated since the last published reviews in 2012, 2013, and 2016. Three types of reactions continue to be studied: glycerolysis of urea, transcarbonation of DMC, DEC, or cyclic carbonates with glycerol and reaction using CO_2_. Among these different routes, DMC and glycerol were selected as the raw materials for the GC synthesis in this work since the transcarbonation from these green reagents leads to high yields and selectivities, using mild conditions including a less energy consuming GC separation process. Catalytic conditions using Na_2_CO_3_ seem to be a good compromise to achieve a high yield of GC, leading to an easier purification step without GC distillation. Mild temperatures for the reaction (73–78°C) as well as a low waste amount confirmed by the E-factor calculation, are in favor of controlled costs. Plasticizing properties of synthesized GC were compared to the behaviors of a commercial plasticizer and natural dialkyl carbonates, for a colorless nail polish formulation. The resulting films subjected to mechanical and thermal stresses (DMA and Persoz pendulum) showed the high plasticizing effect of GC toward nitrocellulose based films, probably due to hydrogen bond interactions between GC and nitrocellulose. The GC efficiency gives the possibility to decrease the content of the plasticizer in the formulation. Glycerol carbonate can be thus considered as a biobased ingredient abiding by the green chemistry concepts, and safe enough to be used in an ecodesigned nail polish formulation.

## Introduction

The global warming of our planet is a major danger for humanity and its environment. Greenhouse gas emissions are the primary cause of this climate change and far from being reduced increases year by year because of the growing of the population. Consequently needs in both food and industrial development regularly increase. In this context, solutions emerge to replace fossil energy sources with renewable energies that are more respectful of the environment. Especially in the transport sector, the biodiesel obtained from plant resources has been developed as a fuel in substitution for traditional petroleum energies. Over the last twenty years, the level of diester production in many countries has led to a significant production of glycerol (GL) as by-product of the process. This crude glycerol is thus produced in large quantities and available on the market at a lost cost. Among the twelve platform chemicals recommended by the US Department of Energy, glycerol is the fastest growing industrial molecule (Averous et al., 2017)^1^.

Glycerol is particularly interesting as a building block to produce bio-based molecules with added value. In the chemicals market, the production of bio-based products has become a priority in the industries of plastics, lubricants, solvents and surfactants: an increase is expected at a CAGR (compound annual growth rate) of 16.67% over the forecast period of 2019–2027 (Global bio-based chemicals market forecast, 2018). Indeed, the manufacture of bio-based molecules offers a good alternative/opportunity to reduce the dependence on petroleum, to improve the environmental balance due to the reduction of CO_2_ emissions, to meet the growing of consumers demand for safer and healthier products and for companies, to set themselves apart from the competition.

**Figure 1:**
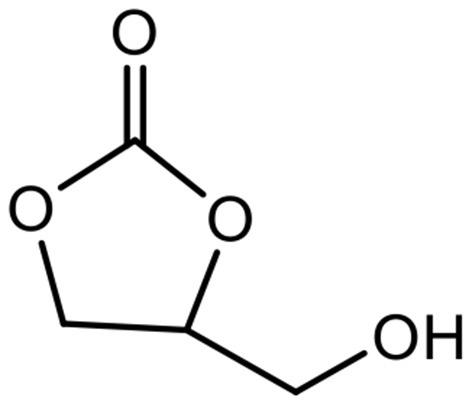
Structure of the glycerol carbonate (GC).

**Figure 2:**
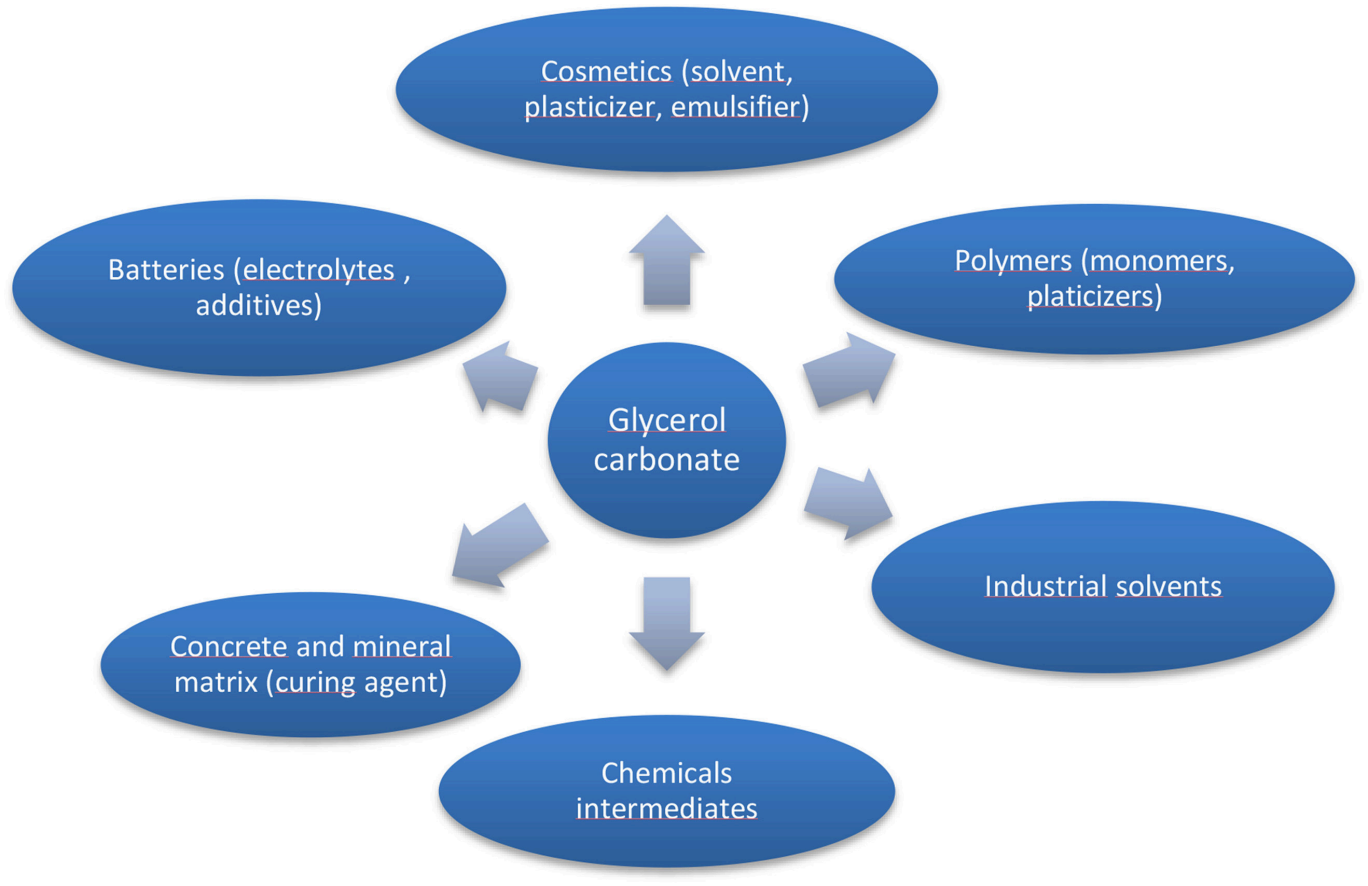
Applications of glycerol carbonate (adapted from references Sonnati et al., 2013; Algoufi et al., 2017).

Among the molecules derived from glycerol, glycerol carbonate (GC) (Figure 1), is a five membered cyclic carbonate remarkable for its physical and chemical properties as it gets an interesting chemical reactivity thanks to its two different functions (hydroxyl and cyclic carbonate). Indeed it can be used as a platform molecule to lead to others value-added products which find applications in a wide variety of fields such as cosmetics, detergents, intermediates for chemistry and polymer synthesis as illustrated in Figure 2. GC is chemically stable, non-flammable (Flash Point >204°C), water-soluble and presents ecofriendly properties: low toxicity (no R-phrase in its material Safety data Sheet), good biodegradability, very low volatility (bp 110–115°C at 0.1 mmHg) and a high renewable content ranging between 76 and 100% depending on its synthesis route (Clements, 2003).

**Figure 3:**
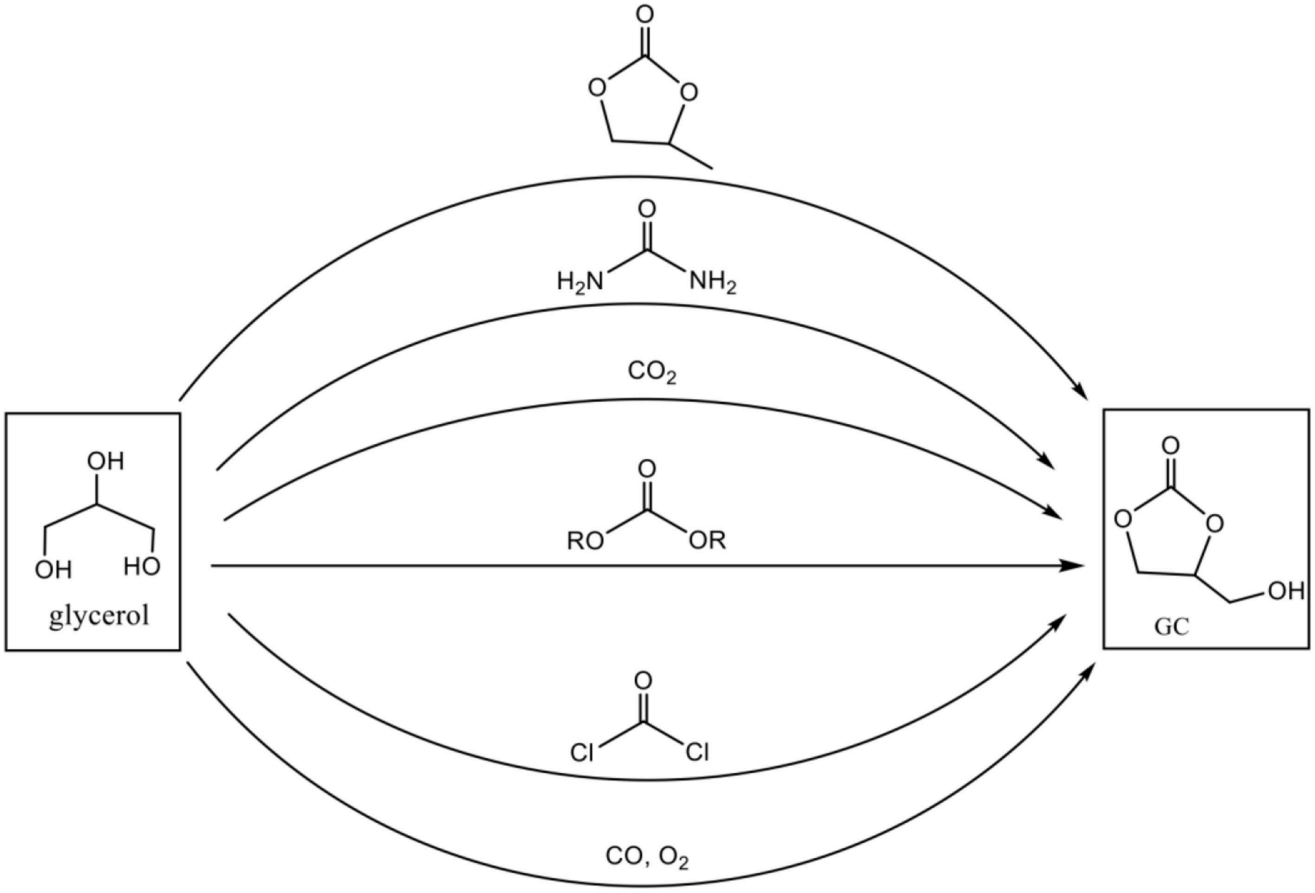
Different conversion routes for synthesis of glycerol carbonate (adapted from reference Van Mileghem et al., 2018).

The development of synthetic routes for obtaining GC follows in parallel the growing interest for its applications (Sonnati et al., 2013). Several conversion schemes exist (Figure 3), involving various reagents as a carbonyl source such as phosgene, carbon monoxide, urea, carbon dioxide, or organic carbonates. However, some of these processes are limited because of the toxicity of the reagents and difficulty to implement the chemical reactions, as it is the case for phosgene and carbon monoxide. So in this contribution we provide an overview on the synthesis of glycerol carbonate based on the three main routes: carbon dioxide, urea and organic carbonates including dimethyl carbonate. A focus on the green aspects of the reactions, their industrial feasibility according to the raw material (crude glycerol obtained directly from biodiesel plants or pure glycerol) and an example of one glycerol carbonate application as plasticizer is also given.

## Materials and Methods

### Chromatographic Analysis

For the GC synthesis, the composition of the reaction mixture was analyzed by HPLC, Spectra System P 1500 Spectra-Physics Analytical. The injector system was Spectra System AS 3000 Spectra-Physics Analytical and the detector was a refractometer (Varian Prostar Model 350 RI detector). The column was apolar (Car-H). The following conditions were used: an eluant corresponding to an aqueous solution of H_2_S0_4_ (0.004 N), a flow at 0.80 mL/min, a column temperature at 35°C and an injection volume of 20 μL.

For the dialkyl carbonates synthesis, the composition of the reaction mixture was analyzed by GC (Chrompack 9002). An on-column injector and a FID detector were used with a polar column CP WAX 25 m^*^0.32 mm^*^1.2 μm. Samples were diluted 1,000 times in acetone.

### Synthesis of Glycerol Carbonate

Dimethyl carbonate (DMC) (45 g, 0.5 mol), 15.3 g glycerol (0.167 mol) and 0.053 g Na_2_CO_3_ (5.10^−4^ mol) were introduced simultaneously into a 250 mL three-neck flask equipped with a condenser, a temperature sensor and a mechanical stirring system. The mixture was stirred and refluxed (75°C). After 2 h of reflux, the conversion rate of glycerol, measured by HPLC, reached 98%. After cooling the reaction medium, the catalyst was filtered off on Buchner and the excess of dimethyl carbonate and the methanol formed were distilled under atmospheric pressure. The glycerol carbonate was obtained as a colorless viscous liquid (19.7 g) with a purity of 98%.

^1^H NMR (400 MHz, CDCl_3_): δ (ppm) = 5.29 (t, 1H, OH), 4.82–4.77 (m, 1H, CH), 4.49 (dd, 1H, OC*H*_2_), 4.29 (dd, 1H, OC*H*_2_CH), 3.66 (ddd, 1H, C*H*_2_OH), 3.49 (ddd, 1H, C*H*_2_OH).

### Synthesis of Dialkyl Carbonates

In a 250 mL three-neck reactor connected to a long distillation column and equipped with a mechanical stirrer and a thermometer, were added 10 g of DMC (10.111 mol), 49 g of isoamyl alcohol (0.555 mol), and 5 g K_2_CO_3_. The mixture was heated for 4 h at 140°C corresponding to a high reflux in the reactor. A mixture of DMC-methanol azeotrope of mass composition 30–70 was recovered at the top of the distillation column (*T* = 63–64°C). The kinetic of the reaction was followed by gas chromatography and the reaction was stopped when DMC has totally reacted. The diisoamyl carbonate was obtained with a selectivity of 99% relative to the intermediate carbonate. A distillation under reduced pressure (92–94°C, 3.8 mmHg) led to diisoamyl carbonate (17.44 g, overall yield 76%) in the form of a colorless and odorless liquid.

^1^H NMR (400 MHz, CDCl_3_): δ (ppm) = 4.11–4.12 (t, 2H, OC*H*_2_), 1.69–1.71 (dd, H, CH), 1.55 (t, 2H, OCH_2_C*H*_2_), 0.90–0.92 (d, 6H, CH_3_).

### Synthesis of Didodecyl Carbonate

In a 250 mL three-neck reactor connected to a cooler, equipped with a mechanical stirrer and a thermometer, were added 15 g of DMC (0.167 mol) and 93.2 g of dodecanol (0.5 mol) and 5 g of K_2_CO_3_. The mixture is heated for 5 h at reflux of DMC. These conditions led to the symmetrical carbonate with a selectivity of 85% relative to the mixed carbonate. The purification is carried out by distillation under reduced pressure (180°C, 1 mbar) to distill only the dodecanol and recover a mixture of composition (dilauryl carbonate: methyl lauryl carbonate 94: 6). 33.3 g of dilauryl carbonate (48%) is obtained as a viscous colorless liquid.

^1^H NMR (400 MHz, CDCl_3_): δ (ppm) = 4.12 (t, 2H, OC*H*_2_), 1.55 (t, 4H, OCH_2_C*H*_2_), 1.26–1.32 (m, 18H, CH_2_), 0.87–0.90 (t, 3H, C*H*_3_).

### Pendulum Hardness Test

Persoz hardness is measured with a pendulum of Persoz according to the protocol that meets the standards ASTM D 4366 and EN ISO 1522. The apparatus used is an ERICHSEN Pendulum Damping Tester model 299/300. Oscillations of a pendulum in contact with the tested surface have a damping time directly proportional to the hardness of this surface.

A 300 μm wet film was applied to a glass plate. The measurements were made at regular intervals (30 min, 1, 2, 4, 24 h) after drying the varnish film in a thermostatically controlled chamber at 20°C. The plasticizing properties of a molecule is therefore inversely proportional to the number of oscillations made by the pendulum.

### Dynamical and Mechanical Temperature Analysis (DMA)

The sample was prepared by pouring the formulation into a teflon matrix and then dried on a plate thermostated at 30°C for 24 h under room humidity. Specimens (200 μm thick, 10 mm wide and 7 mm long) were cut from the resulting molded film. A pre-stressing of 0.5 N was imposed on the sample before the beginning of the measurement in order to put the film under tension. Tensile tests and small deformations were applied at the 1 Hz frequency while imposing a sinusoidal displacement of 8 microns. The sample was subjected to a temperature sweep (3°C/min) over the range −50 to 60°C.

## Research Update on GC Synthesis

### Synthesis of GC From Glycerol and CO_2_

The use of CO_2_ as C1 chemical feedstock to prepare useful products such as organic carbonates, methanol and polycarbonates on a large scale would be ideal for industrial applications, resulting in the consumption of million tons of CO_2_ per year (Dabral and Schaub, 2019). Performing CO_2_ conversion is a great promise for recycling CO_2_, as only a small proportion of the total abundance is currently being consumed by the chemical industry (Song Q. W. et al., 2017). Therefore, preparation of GC directly from glycerol and CO_2_ is an ideal strategy for dealing with these two by-products, commercially available at low price. The atom efficiency of the reaction could be high if we consider H_2_O as the only side product. Thus, much attention has been focused on this reaction which is in line with the concept of green and sustainable development.

The direct carbonylation of glycerol with CO_2_ was carried out in homogeneous and heterogenous catalysis. The first experiments in presence of metal alkoxides such as n-Bu_2_Sn(OMe)_2_ and n-Bu_2_SnO, were done using various experimental conditions (Aresta et al., 2006). Unfortunately, the reaction was unsuccessful with low conversion of glycerol due to the low CO_2_ reactivity. Moreover, the original catalysts were converted into oligomers showing a moderate catalytic activity. When the same reaction was carried out in methanol as solvent, the yield was increased to 35% (George et al., 2009). Following these poor results and the thermodynamic stability and kinetic inertness of CO_2_, solid metallic oxides were used for their potential activity in the transformation of CO_2_ due to the surface adsorption and activation of CO_2_.

**Figure 4:**
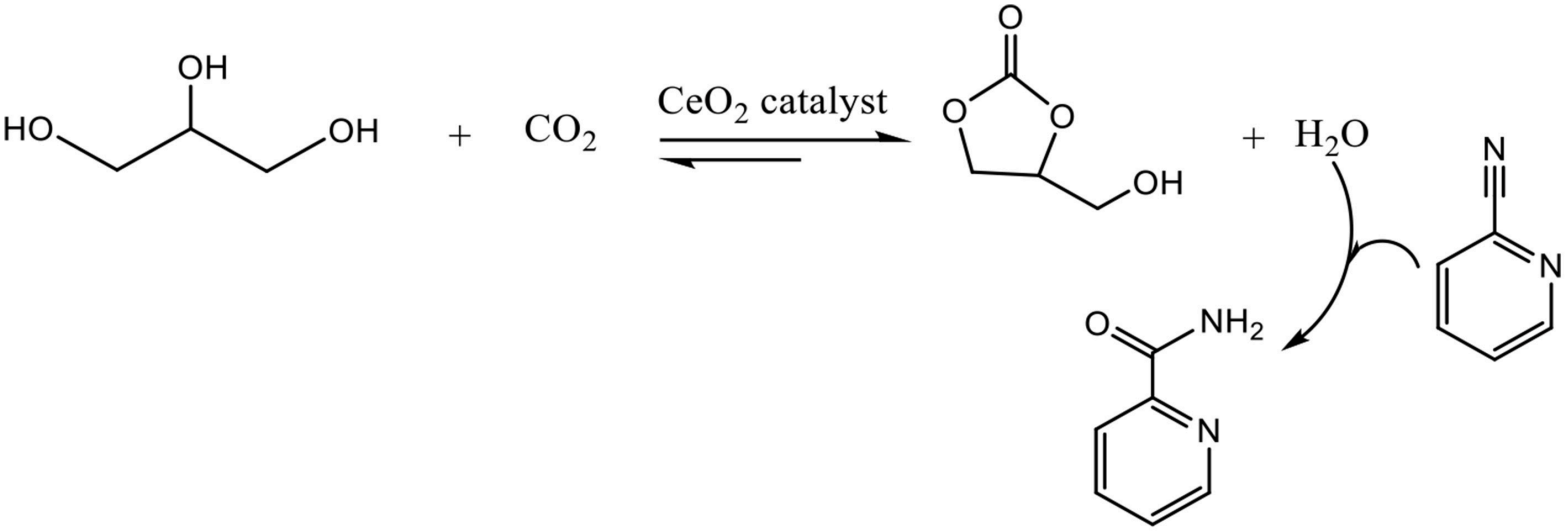
Reaction of glycerol and CO_2_ with CeO_2_ in presence of 2-cyano-pyridine.

CeO_2_ nanoparticles or nanorods were employed as catalyst in the presence of 2-cyanopyridine, which was used as a dehydration reagent to remove water and shift the chemical equilibrium to the glycerol carbonate side (Figure 4) (Liu et al., 2016). 2-cyanopyridine reveals to be a better dehydrant than acetonitrile which can gives the formation of by-products with the resulting hydrolysis product and glycerol (Li et al., 2015). Moreover, the hydration of the resulting amine into acid does not occur over CeO_2_ catalysts resulting in a higher selectivity toward GC (Liu et al., 2016).

The system showed good catalytic performances in the carbonylation of glycerol and CO_2_ with the efficient hydration of 2-cyanopyridine and solvent effect of DMF, obtaining a 21% yield of GC. The redox properties of CeO_2_ played an important role on the activity. The reaction conditions were 3 times the stoichiometric value of 2-cyanopyridine, 150°C, 4 MPa and 5 h. The catalyst could be easily recycled through the calcination process at 400°C for 5 h, and the activity of the regenerated catalyst stayed constant after recycling for five times. To enhance the catalytic performances, Zr doped CeO_2_ was used and the influence of molar ratio of Zr/Ce on the crystalline structure, surface composition, redox and acid-base properties were studied (Liu et al., 2018). The conversion of glycerol increases to 40% and the yield of GC to 36.3% in presence of a 0.02 molar fraction of Zr. This good performance is explained by the acid-base properties of the catalyst, knowing that the Lewis acid sites are appropriate to the absorption and activation of OH group of glycerol and the basic sites favor the CO_2_ absorption and activation.

**Figure 5:**
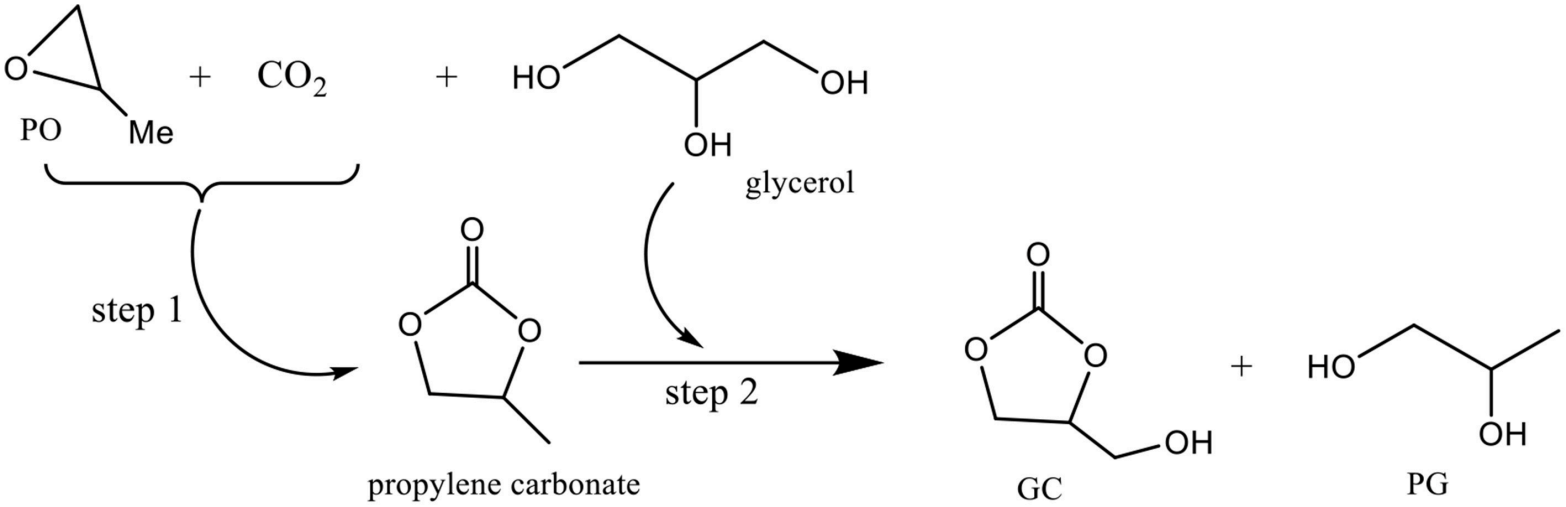
Reaction of glycerol in presence of CO_2_ and PO.

Besides acetonitrile and 2-cyanopyridine introduced as chemical dehydrating agents to eliminate the formed water, 13X zeolite and molecular sieves were used as physical dehydrating agents to absorb water but the results were unsatisfactory (George et al., 2009). New strategies have emerged for the carbonylation of glycerol with CO_2_. Pioneering work on the preparation of GC using KI as a highly efficient homogeneous catalyst and propylene oxide (PO) as a coupling agent to make the reaction thermodynamically favorable has been published (Ma et al., 2012). The coupling reaction between PO, glycerol and CO_2_ consists of a cycloaddition of CO_2_ with PO (step 1, Figure 5) and a subsequent transesterification of glycerol with carbonate to form GC and propylene glycol (PG) (step 2, Figure 5). The simultaneous conversion of glycerol and CO_2_ via the coupled reaction can be carried out very effectively using KI as the catalyst since 78% glycerol conversion was achieved with a yield of 40% glycerol carbonate. Despite the good catalytic performance of KI, its usage is limited due to the difficulty in separating it from the reaction system.

Therefore, an efficient strategy for developing a heterogeneous, reusable and easily recoverable catalyst from the reaction system has been studied (Song et al., 2018). A functionalized heterogeneous catalyst was developed by introduction of imidazole-based ionic liquids into the structure of a DVB-based polymer showing, in presence of PO, a satisfying activity toward both the coupling of PO with CO_2_ and the transesterification of PC with glycerol could be achieved, implying the high catalytic performance of ionic liquid groups in the coupling reaction of PO with CO_2_. The conversion of PO reached up to 96% with a GC yield of 81% in presence of the prepared PDVB-(vIm-BuBr) catalyst. Moreover, this latter showed excellent stability during the reaction, and maintained its catalytic activity after five cycles. The high activity and remarkable stability of this heterogeneous polymer P-DVB-(vIm-BuBr) catalyst represent interesting features for future industrial applications.

### Synthesis of GC From Glycerol and Urea

**Figure 6:**
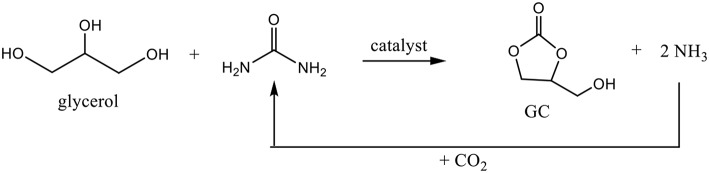
Reaction of glycerol in presence of urea and urea synthesis from NH_3_ and CO_2_.

Beside the direct synthesis of GC from glycerol and CO_2_, the exploration of another indirect synthesis approaches using urea as a CO_2_ donor may simplify the glycerol production and may lead to a more economical process (Claude et al., 2000; Nguyen and Demirel, 2013). Urea glycerolysis represents an attractive alternative method to produce GC because it is a biobased reactant containing an activated form of CO_2_. The reaction was run under a reduced pressure in order to remove the ammonia formed during the reaction and move the equilibrium to a maximum conversion. Furthermore, ammonia formed can be easily converted to urea since urea synthesis is performed from ammonia and carbon dioxide (Figure 6).

Heterogeneous catalysts have been developed and they have been particularly efficient for carbonylation of glycerol with urea affording good yield under moderate reaction conditions. Recently the carbonylation of glycerol with urea was reported at 145°C using MgO and CaO basic oxides, Al/Mg and Al/Li mixed oxides derived from hydrotalcites with adequate acid–base pairs. About 72% of glycerol carbonate yield was achieved in 5 h of reaction time with 82% of glycerol conversion (Climent et al., 2010). The results showed that catalysts exhibiting adequate acid-base properties were efficient to the synthesis of GC. The effect of Zr doping on the acidic-basic properties of calcined Mg-Al hydrotalcite and their catalytic performance for the GC synthesis have been demonstrated as a 85% yield of GC could be obtained with a glycerol conversion of 97% and a selectivity of 90%. The Zr content could affect significantly the acidic-basic property (Wan D. et al., 2018).

The impact of these acidic and basic properties had already been shown in the study involving γ-zirconium phosphate catalyst exhibiting about 80% glycerol conversion with 100% selectivity toward GC (Aresta et al., 2009). The presence of Lewis acid sites able to activate the carbonyl group of urea makes the nucleophilic attack of the glycerol absorbed on the Lewis basic sites easier. The reaction mechanism describing the role of the basic and acidic active sites is proposed by Fernandes and Yadav (2013) based on their experiment observations and the effect of different parameters during their study on the glycerolysis using combustion synthesized magnesium oxide as catalyst. A well-balanced acidic-basic property was important to obtain high GC yield with good selectivity.

A series of tin-tungsten mixed oxides with different Sn to W molar ratio were synthesized and characterized by various spectroscopic techniques and were involved in the carbonylation of glycerol (Jagadeeswaraiah et al., 2014). The catalysts were active for selective formation of glycerol carbonate and the best one was the catalyst Sn/W ratio 2:1 calcinated at 500°C giving 52% glycerol conversion with >99% GC selectivity. The catalyst without any pre-treatment was recycled with a consistent activity.

An interesting study on a green co-generation of high purity of zinc glycerolate and glycerol carbonate was published using glycerol as reactant and as a raw material to prepare the catalyst (Zhang et al., 2016) The zinc glycerolate obtained by mixing Zn acetate with glycerol was decomposed to ZnO by calcination. ZnO was highly active toward the reaction, yielding 85.97% of GC. The study revealed that the ZnO catalysts have the advantages of high activity, high recyclability, high stability, and environmental friendliness that can be appropriately applied in industrial settings.

The dual catalysis over ZnAl mixed oxides consisting of ZnO and ZnAl_2_O_4_ phases was investigated. The ZnAl mixed oxides showed much higher glycerol conversion and GC yield than the ZnO and ZnAl_2_O_4_. The ZnO phase provided a homogeneous reaction route via the dissolved Zn species, resulting in the zinc isocyanate Zn(NCO) complex formed in the liquid phase suggested as a main active site (Fujita et al., 2013). The Zn(NCO) complex also adsorbs on the ZnAl_2_O_4_ phase and acts as a supplementary heterogeneous active site, in addition to the original Lewis acid-base active site of ZnAl_2_O_4_. The combination of these homogeneous and heterogeneous reaction routes enhances the catalytic performance of the ZnAl mixed oxide (Nguyen-Phu et al., 2018).

Despite these many promising results achieved in the study in the urea glycerolysis, most of the works either utilized commercial glycerol as a starting material. Indeed the main reason is due to the presence of impurities in the crude glycerol which can affect the transformation of glycerol into the targeted value-added product. Few studies have been reported on the production of GC through the direct use of crude glycerol. Recently, an economical and green synthesis approach using boiler ash (BA) from palm oil industry as catalyst for the reaction of producing GC from crude glycerol and urea, has been studied (Paroo Indran et al., 2014). It was the first example on the use of waste material, containing many metals of those being major are potassium, calcium, and magnesium, as catalyst for the production of GC. Various catalysts calcinated at different temperatures showed relatively comparable glycerol conversion of about 90%. The potassium silicate BA 900 (calcination at 900°C) gave the highest GC selectivity and yield of 90 and 84.3% respectively. The authors explained this result by the presence of potassium ion (higher metal content with a percentage of 86%) which acts as a weak Lewis acid to catalyze the reaction. Moreover, the presence of oxide ion (O^2−^) certainly plays a role of strong base to activate glycerol to form GC. In a similar study, boiler ash containing BA 900 and potassium silicate (K_2_SiO_3_) were proven to be feasible Lewis acid catalysts for the synthesis of GC. The catalytic system was reusable for three consecutive reaction cycles without the loss of activity (Indran et al., 2016). In another study, the same catalytic system was involved in the glycerolysis of crude glycerol from two different sources obtained from the commercial biodiesel plant depending on the catalyst used either sodium methylate or potassium methylate (Paroo Indran et al., 2017) The results confirmed the activity of the catalytic system but the selectivity and yield C are lower than those obtained using commercial glycerol. The water and methanol present in the crude glycerol, even in small quantities, are responsible, knowing the instability of GC in water and methanol can cause decomposition of GC into glycidol (Teng et al., 2014). Nevertheless, the current results suggest that the crude glycerol can be directly transformed using boiler ash as catalyst, even if the optimal yield of GC cannot be obtained. This study is interesting because it represents an example of a totally green synthesis approach.

### Transcarbonatation of Alkyl or Cyclic Carbonate With Glycerol

#### Chemical Catalysis

**Figure 7:**
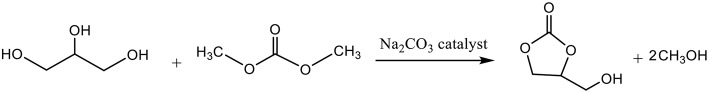
Transcarbonatation of glycerol with dimethyl carbonate.

Glycerol carbonate (GC) can also be synthesized by transesterification of cyclic carbonates (most often ethylene or propylene carbonate) or aliphatic carbonates (dimethyl carbonate or diethyl carbonate) (Figure 7) with glycerol in the presence of a basic catalyst, the acid catalysts having proved to be slightly effective (Teng et al., 2016). The addition of solvent is not necessary since the carbonates, reactants in excess of glycerol, also play the role of solvent in these transcarbonation reactions. This excess (carbonate:glycerol molar ratio generally between 2:1 and 5:1) makes it possible to shift the reaction equilibrium toward GC synthesis: this method is more effective than the addition of molecular sieves to remove the methanol formed (Lanjekar and Rathod, 2013).

Numerous synthesis conditions of this transcarbonation reaction have been compared in the reviews of Ochoa-Gómez et al. (2012); Sonnati et al. (2013), and Teng et al. (2016), and in particular, the basic catalysts. For transesterification reactions from dimethyl carbonate DMC or diethyl carbonate DEC were tested CaO, MgO, K_2_CO_3_, CaCO_3_, KOH, NaOH, K_2_CO_3_/MgO, Mg/Al/Zr mixed oxides, Al/Mg hydrotalcites, KF-hydroxyapatite, lipases and ionic liquids including 1-n-butylimidazole (Naik et al., 2009). It is important to point out that this latter catalyst allows to convert efficiently crude glycerol (purity of 88 wt%–contaminants: water and traces of alkaline salts) while most of the syntheses have been studied from pure glycerol or containing < 2% water (Rokicki et al., 2005). Water can cause deactivation of catalysts such as extruded CaO/Al2O3 (Lu et al., 2013). However, according to Bai et al. (2011), the KF-hydroxyapatite-based catalyst may not be degraded by the presence of water and soap, which suggests that synthesis of GC from crude glycerol could be achieved without the need to implement an expensive distillation process after the production of biodiesel.

For reactions from ethylene carbonate (EC), the catalysts CaO, Al/Ca mixed oxides, Li-hydrotalcite, basic resins of the type Amberlyst A26 (${\text{HCO}}_{3}^{-}$), immobilized ionic liquids have been studied (Ochoa-Gómez et al., 2012; Sonnati et al., 2013; Teng et al., 2016). The results have shown that regardless of the homogeneous or heterogeneous basic catalyst tested, conversions of glycerol and reaction selectivities are high: conversions are >95% from DMC and >85% from EC and selectivities are generally ranged between 95 and 99% from DMC and between 84 and 99% from EC. According to (Ochoa-Gómez et al., 2012), the transcarbonation route from DMC or EC with glycerol is suitable for industrial production and involves uncalcined CaO as this heterogeneous catalyst is cheap and can be easily separated by filtration. Although its performance is slightly lower than that of the calcined catalyst due to the presence of impurities such as Ca(OH)_2_ and CaCO_3_, the calcined catalyst is deactivated and must be regenerated after reaction.

Although the reaction temperatures used are lower for the transcarbonation of EC with glycerol (35–80°C vs. 75–120°C for the reaction with DMC) due to a decrease in the constants of chemical equilibrium as a function of temperature (Li and Wang, 2011), DMC could be preferentially selected industrially (Hirotsu and Kaneko, 2001; Hérault et al., 2003) because the purification step of the GC is less energy consuming. Indeed, the boiling temperatures of DMC (90°C) and methanol (65°C) are much lower than those of EC (261°C) and ethylene glycol EG (197°C). In addition, reactions with DMC are usually carried out at atmospheric pressure while, for reactions from EC, a reduced pressure (35 mmHg) is often applied to remove EG and displace reaction equilibrium.

Since 2014, the date of the last review on the synthesis of glycerol carbonate, other work about transcarbonatation with glycerol has been published and highlight the strong catalytic activity of other bases such as:

- The calcined silicates (Wang et al., 2017) (Na_2_SiO_3_) that can be reused 5 times without significant decrease of its catalytic activity,
- Li doped La_2_O_3_ (Li Y. et al., 2018) or ZnO (Song X. et al., 2017) or Mg_4_AlO_5.5_ (Liu et al., 2015). Li doping was shown to improve the catalytic activity due to its positive effect on the basic properties and the interaction between Li and the metal oxide supports.
- Other mixed oxides catalysts such as Ce-NiO (Wu et al., 2017), CeO_2_-CdO (Wu et al., 2018), Sr/Al mixed oxides and in particular the catalyst With Sr/Al ratio of 0.5 (Algoufi et al., 2017): high reaction selectivity (91–100%) and glycerol conversion (94–97%) were also obtained for the GC synthesis from diethyl carbonate (instead of DMC) for the two former catalysts.
- Basic resins such as Ambersep® 900 (hydroxide functional resin) filling glass column reactor in a continuous flow process. High glycerol conversion and reaction selectivity with a short residence time (10 min) are resulted from this process (Van Mileghem et al., 2018).
- DABCO embedded porous organic polymer (Wan Y, et al., 2018): the efficiency of this catalyst can be explained by its porous structure and its amphiphile characteristic which is supposed to make a miscible microenvironment while reactants are immiscible. It has been reused up to 13 times without obvious deactivation.

All the studies using all heterogeneous catalytic materials previously mentioned show the key parameters for a catalyst to be effective in GC synthesis: the strength of basic sites and the porous structure allowing mass transfer and diffusion of reagents to active sites (Okoye and Hameed, 2016).

- Amidines based ionics liquids (Ishak et al., 2017). The results depend on both cation and anion used, knowing that the anion activates hydroxyl groups of glycerol through an hydrogen bonding interaction (Ishak et al., 2016). 1,8-diazabicyclo[5.4.0]undec-7-ene (DBU) based ionic liquid was proved to be the best catalyst for converting 98% glycerol to produce GC with a selectivity of 96% and a high turnover number (TON = 9408 within 7.5 h and 0.01 mol% of catalyst loading) (Munshi et al., 2014a,b).
- Homogeneous ammonium catalysts: tetraethylammonium pipecolinate gives the best results in terms of glycerol conversion and reaction selectivity (Van Mileghem et al., 2018).

#### Biocatalysis

Another catalytic route of glycerol transesterification reactions with DMC to synthesize GC is the biocatalytic route with the use of lipase type enzymes. These reactions under kinetic control lead to high yields according to the experimental conditions. Studies showed (Du et al., 2018) the optimization of the reaction with the immobilized *Candida Antartica* lipase B as biocatalyst at a concentration of 5 g/L, a GL/DMC molar ratio (1:20), at 50°C. After 24 h the yield in GC is 89%. Under these conditions the authors showed that the presence of organic solvents could be harmful to the reaction and worked in a solvent-free system.

Netherless other authors (Jung et al., 2012) relate the positive effect of hydrophilic solvents, such as THF, tert-butanol, and acetonitrile. These solvents would play a favorable role by reducing the coating effect of insoluble glycerol on the surface of the immobilized enzyme. Thus, a maximum yield of 77% could be achieved with the solvent acetonitrile and the surfactant Tween 80 helping the mixing of the reagents. On the other hand, hydrophobic solvents such as hexane, toluene, and xylene did not allow the reaction.

The role of water in the biocatalytic transformation of GL is not so obvious (Kim and Lee, 2018): beyond 0.5% v/v it seems to have a negative effect on the reaction by inhibition of enzymatic activity. However, small quantities of water could play a positive role by improving interfacial availability by preserving the 3D structure of the enzyme in non-aqueous media.

If we switch to the feasibility of achieving the biocatalytic conversion of crude glycerol to GC, the adverse effect of water on enzyme activity by hydrolysis could be effectively reduced through pre-treatment processes, keeping a low amount of moisture favorable to the reaction by improvement of the enzymatic catalytic activity. Thus, prospects for the use of crude glycerol containing traces of water for biocatalyzed reactions (Tudorache et al., 2014; Luo et al., 2016 are opened, whether for the synthesis of GC or other value-added chemicals.

#### Crude Glycerol as Starting Material

The transformation of crude glycerol into hydrogen, polyglycerols, epichlorohydrin, or polyols for polyurethane foam synthesis can be interesting and competitive in comparison with results obtained from pure glycerol (Kong et al., 2016). This added value finds a significant meaning with the esterification or transesterification reactions like in glycerol carbonate synthesis.

During the production of biodiesel, the crude glycerol obtained as a byproduct has a very different composition depending on the technology used or the sourcing of the oils. The glycerol content can vary from 30 to 70% depending on the process and the oil and reaches 80% after acid treatment (Nanda et al., 2014; Kong et al., 2016). The main impurities present which can interfere with the transformation of crude glycerol, are water, methanol, soaps, free fatty acids (FFAs), fatty acid methyl esters (FAMEs), glycerides, and ashes. The economic advantage of the use of crude glycerol has led to a deeper knowledge (Hu et al., 2012) of these impurities and the understanding of their effects on glycerol performance, especially during transesterification reactions.

Studies show that from 10% w/w of water, GC becomes instable and has a significant influence on crude glycerol conversion (Paroo Indran et al., 2017). Similarly, a 5% w/w methanol content interferes with the selectivity of the reaction.

But these conditions are extreme and in reality the crude glycerol may not reach this content of impurities. On the contrary, the potassium and calcium methylate residues resulting of the biodiesel production process, can effectively act as a catalyst in its transformation into GC. In fact, very high conversion and selectivity rates have been demonstrated on PG by a joint effect of Ca and Mg species in the catalyst structure (Zheng et al., 2015).

CaO is a very good catalyst in transesterification reactions and can be easily used in industrial processes. For example, GC synthesis from crude glycerol containing 1% CaO with a 2: 1 ratio of DMC / glycerol is optimized with microwave activation for 5 min at 65°C to give a 93% yield, while under the same conditions the pure glycerol leads to only a 5% yield after 90 min of reaction (Teng et al., 2016). These better performances of crude glycerol transesterification compared with pure glycerol are also shown with conventional heating activation conditions (84 vs. 10%).

## Results and Discussion

### A New Catalyst System for Transcarbonatation of Glycerol

Although in terms of sustainable chemistry, CO_2_ is a reagent of choice for GC synthesis, its reaction with methanol is thermodynamically limited, which leads to relatively low conversions and yields compared to other methods of GC synthesis. As for the glycerolysis process of urea, a reduced pressure in the reactor is necessary to remove the ammonia and shift the equilibrium. In addition, the GC purification process coupled to this reaction tends to be the most complex because it requires a multistep GC purification process (liquid-liquid extraction, evaporation and then vacuum distillation).

In this work, we preferred to use the DMC for the GC synthesis because the transcarbonation involves mild conditions and leads to high yields and selectivities. We can also expect the GC separation process to be less energy consuming than the one using cyclic carbonates because of the higher boiling points of cyclic alkylene carbonates and byproducts (glycols).

To test the properties of glycerol carbonate as a plasticizer for a nail polish, glycerol carbonate has been synthesized by transcarbonatation with DMC, with a heterogeneous catalyst.

Unlike K_2_CO_3_, the catalysts CaO and Na_2_CO_3_ are insoluble in the hot medium composed of DMC, glycerol and glycerol carbonate. Note that a supported catalyst K_2_CO_3_/MgO prepared by dry impregnation was also tested with good results (Du et al., 2012).

It was found that 3 wt. % of CaO (relative to glycerol weight) added to a DMC to glycerol molar ratio of 3:1, gave a GC yield of 92% after 2 h, a yield measured without isolating the synthesized GC (Roschat et al., 2018). When the reactant molar ratio was 1:1, an azeotropic agent (benzene) was used to remove continuously the produced methanol during the reaction (Li and Wang, 2010). In these conditions, it was possible to reach a GC yield of 98% with a CaO: glycerol molar ratio of 2%.

Another work (Li W. et al., 2018) proposed the perevaporation as an alternative technology to purify the final medium resulting from transesterification between glycerol and dimethyl carbonate. It was thus possible to separate the four components of the mixture (methanol as a by-product, DMC in excess, non-reacted glycerol and GC) and to break the methanol:DMC azeotrope. However, it was concluded that the selective recovery of methanol remains a future objective.

The reaction was implemented with the selected catalyst, Na_2_CO_3_ (2.9 wt%), with a ratio DM: glycerol equal to 3, under a reflux at 75°C for 2 h. This system showed a great efficiency since glycerol conversion reached 98% (Bandres et al., 2010).

This process presents several advantages compared to usual processes since it generates pure glycerol carbonate (low residual glycerol). Catalyst was recovered by filtration before the distillation of a light fraction composed of MeOH:DMC (azeotrope at 63.5°C).

Calculation of green metrics leads to an atom economy of 65%, an environmental factor of 0.77 and a reaction mass efficiency of 56%. These results can be compared with the performances of same reaction catalyzed by K_2_CO_3_, generating lower green metrics (AE = 43%, RME = 29.5%) and a higher e-factor (E-factor = 2.4). The heterogeneous process using urea and catalyzed by Zn_2_CO_3_, has only improved atom economy (AE = 77%), while E-factor = 1.7 and RME = 37% are once again penalized.

**Figure 8:**
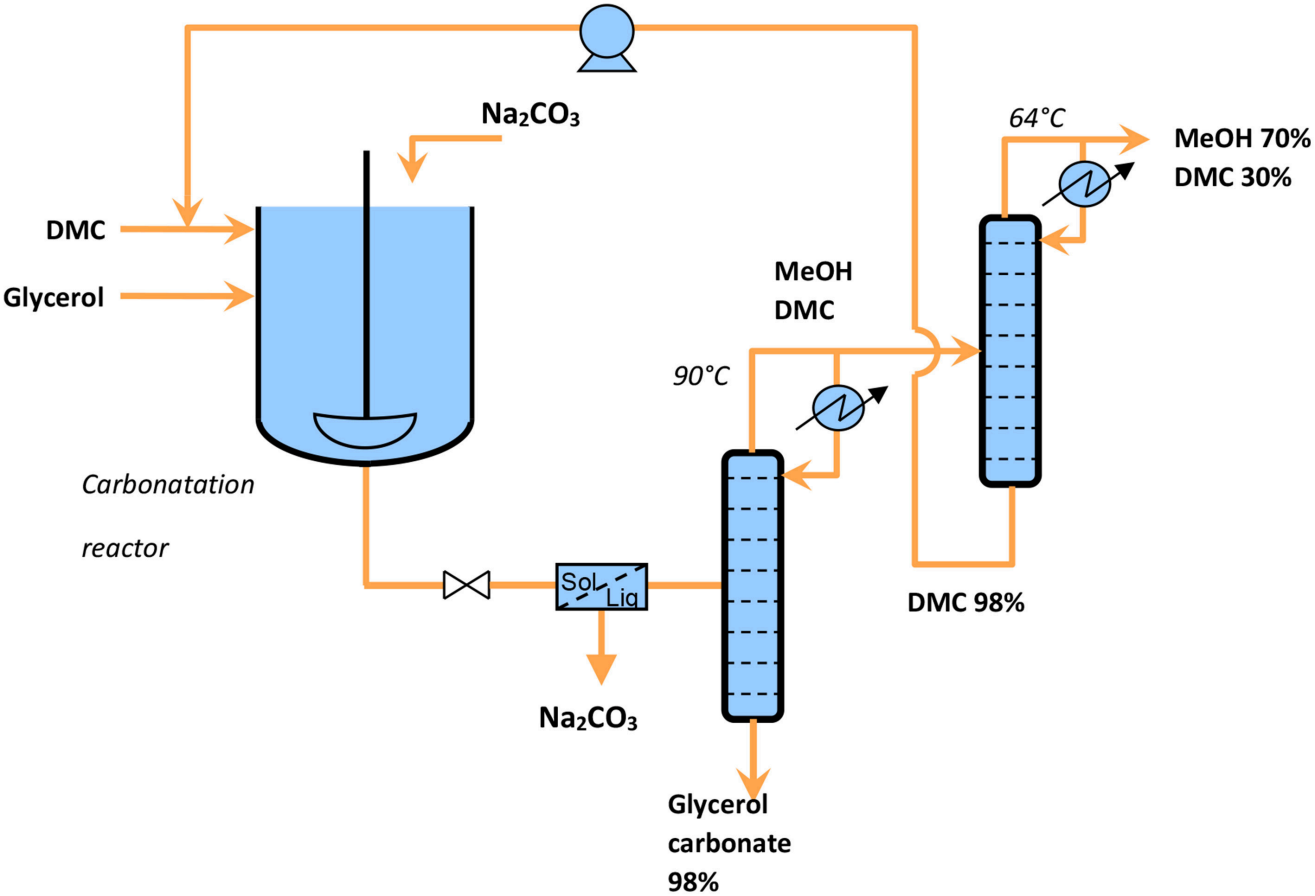
Continuous process proposed for glycerol carbonate production.

For the reaction catalyzed by Na_2_CO_3_, the easy purification and the low production of waste lead to a very interesting E-factor (and a high reaction mass efficiency), conditions which are suitable for scaling-up. The collected data allow to design a continuous process as shown in Figure 8. The reactor outlet flow analyzed by HPLC, is composed of glycerol (0.5%), glycerol carbonate (32.1%), dimethyl carbonate (50.1%), and methanol (17.3%). The second distillation column allows to separate the excess of DMC to recycle it as a reactant. GC collected at the bottom of the distillation column was a viscous, colorless liquid with a purity of 98%. Moreover, these operating conditions, in particular in terms of reduced wastes and moderate temperatures, seem to meet the technical and environmental requirements expected by industry.

### Assessment of GC Plasticizing Properties

Glycerol carbonate was already used as a plasticizer of polymer electrolyte (Yuan, 2015). During the polymerization, glycerol carbonate was added to P(AN-MMA) to form the gel electrolyte. The effect of the plasticizer on the conductivity of the copolymer electrolyte was then studied. Glycerol carbonate was also mentioned in a composition for plasticization of aliphatic and aromatic polyesters (Wypych, 2017).

Our work dealt with the study of plasticizing effect of the synthesized glycerol carbonate within a nitrocellulosic film. Glycerol carbonate was incorporated in a nail polish formulation as an additive (5.6%). The properties of different organic carbonates including natural carbonates are compared with a commercial plasticizer (acetyl tributyl citrate) taken as a standard. Table 1 indicates the composition of the formulation used to prepare the film.

**Table 1:** Compositions of colorless nail polishes.

**Ingredients**	**Composition 1 (%)**	**Composition 2 (%)**
Solvents^*^	62.6	59.1
Isopropanol	14.4	–
Nitrocellulose	10.2	17.9
Polyester resin	7.2	13.3
Plasticizer	5.6	9.9

* *A mixture of ethyl acetate, butyl acetate and isopropanol*.

Glycerol carbonate was perfectly solubilized in the formulation and the resulting film was transparent, unlike the film obtained with dilauryl carbonate which tends to whiten.

The film samples prepared using composition 1 were analyzed by Dynamic Mechanical Analysis. Under the sinusoidal mechanical stress applied to samples, the deformation amplitude of a viscoelastic material is shifted by a phase angle δ.

**Figure 9:**
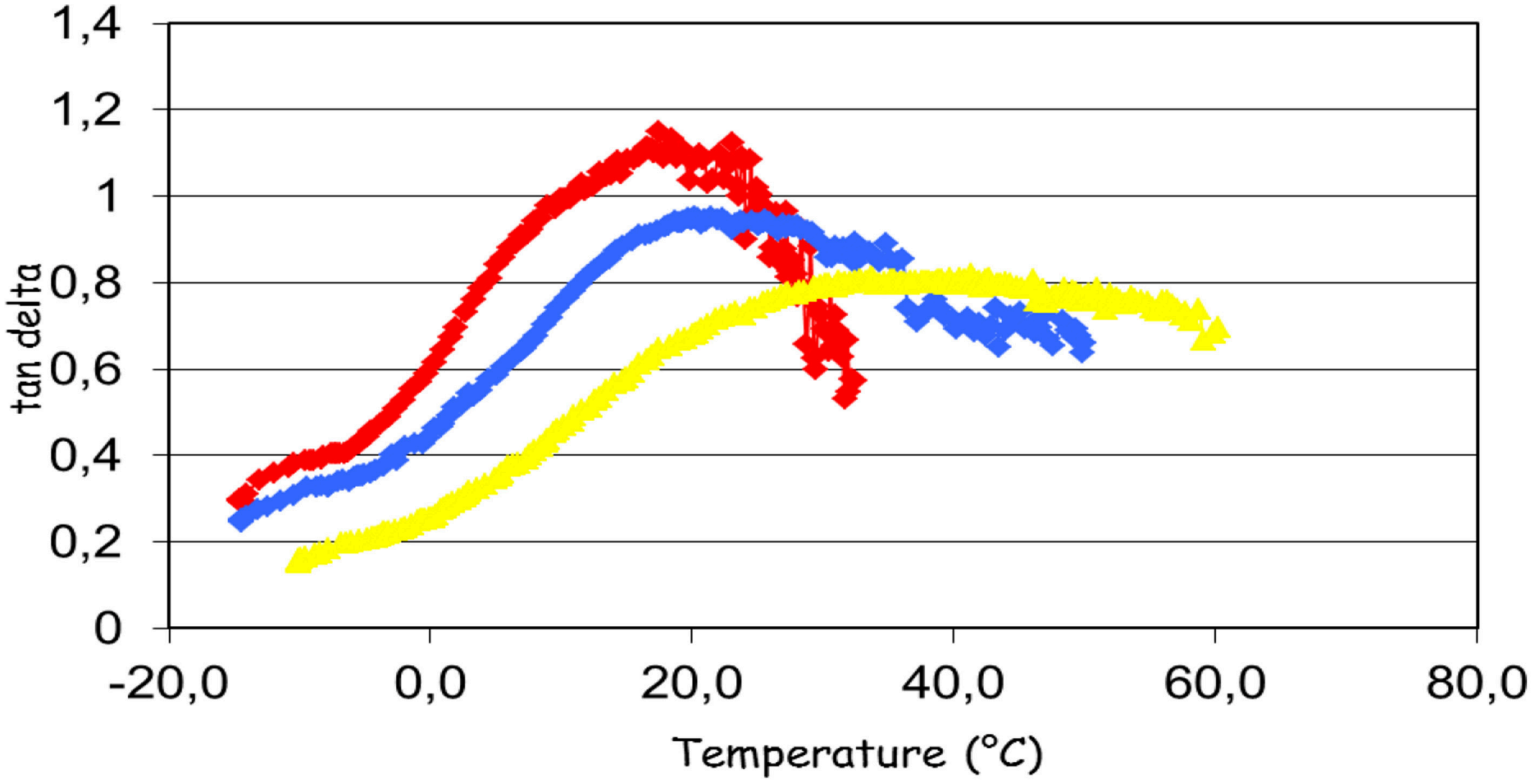
Effect of plasticizers on glass temperature of nitrocellulosic films. Tg = 17.4°C no plasticizer (red line); Tg = 22.3°C with addition of acetyl tributyl citrate (blue line); Tg = 38.7°C with glycerol carbonate addition (yellow line).

Nitrocellulose is a thermoplastic polymer; the behavior of plasticized nitrocellose was studied by Baker et al. (1984). The glass transition temperature of nitrocellulose is close to 40°C, as indicated by the maximum of the tan δ curve as a function of temperature (Figure 9). The reduction of Tg for nitrocellulose plasticized with glycerol carbonate or with acetyl tributyl citrate is distinctly observed. The decrease of Tg reflects the action of a plasticizer. It is also noted that at the same concentration, the plasticizing effect of glycerol carbonate is higher than for citrate. Glass transition temperatures of nitrocellulosic films are presented in Table 2.

**Table 2:** Glass transition temperatures of the nitrocellulosic films according to the plasticizer.

**Plasticizer**	**T_g_ obtained by DMA (^**°**^C)**
No plasticizer	38.7
Diisoamyl carbonate	36.5
Dilauryl carbonate	35.2
Ethylene carbonate	30.9
Propylene carbonate	26.1
Acetyl tributyl citrate	22.3
Glycerol carbonate	17.4

The results show that the glass transition temperature of the film with the glycerol carbonate is lower than Tg of the film with the commercial plasticizer. The other carbonates have variable performances, without achieving the performance of glycerol carbonate. To avoid the formation of a film too soft, a plasticizer must reach a state of equilibrium, that is to say a constant Persoz hardness. The Persoz pendulum is used to study the change of the hardness of the formulations as a function of time (Figure 9). The test was carried out with the composition 2 (Table 1).

**Figure 10:**
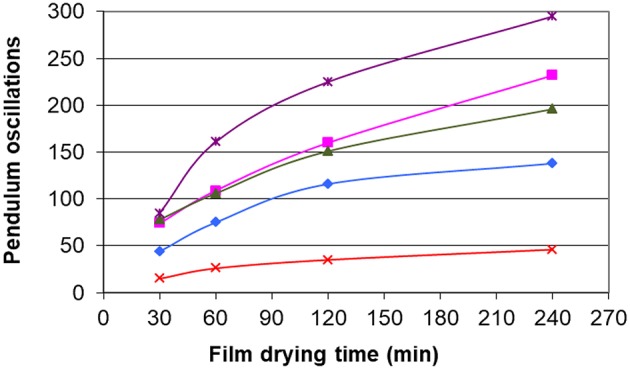
Effect of the plasticizers on the Persoz hardness. ✘ no plasticizer, ■ diisoamyl carbonate, ▲ dilauryl carbonate, ◆ acetyl tributyl citrate, ● glycerol carbonate.

Figure 10 shows that the film containing the glycerol carbonate dampens the oscillations of the pendulum. We can check that the film without plasticizer does not dampen the oscillations. We find that the classification of plasticizers according to their hardness is the same as that obtained with the measurement of the glass transition temperatures. The results thus confirm a moderate plasticizing effect for dilauryl carbonate and diisoamyl carbonate while for glycerol carbonate, plasticizing properties revealed better than those of the commercial plasticizer. Glycerol carbonate content can thus be reduced below 10% to meet the specification of nail polishes.

Finally, results obtained in terms of hardness are in agreement with the decrease of Tg: both approaches show the effectiveness of glycerol carbonate as a plasticizer.

**Figure 11:**
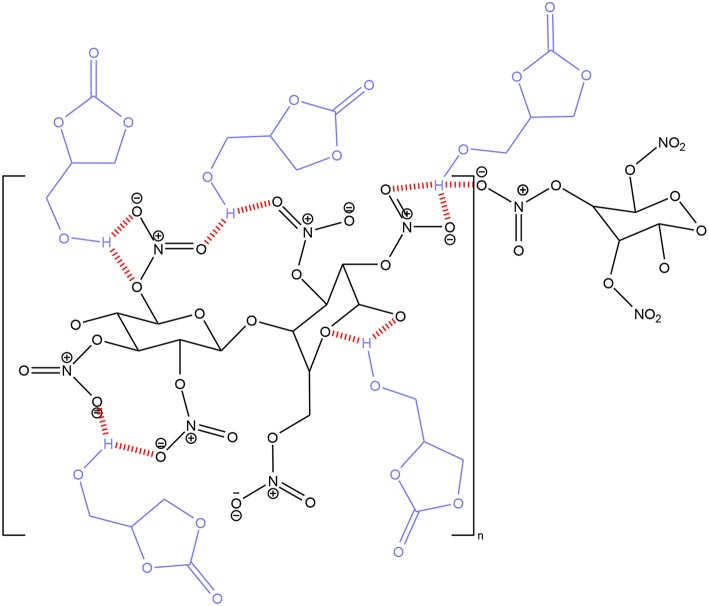
Arrangement of glycerol carbonate molecules within nitrocellulose chains.

These performances suggest that the OH group of glycerol carbonate plays a role in the plasticization of nitrocellulose. Indeed, it can be assumed that the formation of hydrogen bonds between the hydroxyl function and the NO_2_ groups of the nitrocellulose generates an orderly and stable arrangement of the carbonate molecules within the polymeric network, as shown by Figure 11. This characteristic helps to maintain a large and well-distributed free volume between the nitrocellulose chains, thus leading to a high plasticizing effect.

## Conclusion

The state of art on the GC synthesis has been updated since the last published reviews in 2012, 2013 and 2016. Three types of reactions continue to be studied: glycerolysis of urea, transcarbonation of DMC, DEC, or cyclic carbonates with glycerol and reaction using CO_2_. Among these different routes, in this work, DMC and glycerol were selected as the raw materials for the GC synthesis since the transcarbonation from these biobased reagents uses mild conditions including a less energy consuming for GC separation process and leads to high yields and selectivities. Catalytic conditions using Na_2_CO_3_ seem to be a good compromise to achieve a high yield of GC, leading to an easier purification step. Compared to industrial processes, the high glycerol conversion and total selectivity avoid the GC distillation for the application targeted in this work. Mild temperatures for the reaction (73–78°C) as well as a low waste amount confirmed by the E-factor calculation are in favor of controlled costs.

Plasticizing properties of synthesized GC were compared to the behaviors of a commercial plasticizer and natural dialkyl carbonates, for a colorless nail polish formulation. The resulting films subjected to mechanical and thermal stresses (DMA and Persoz pendulum) showed the high plasticizing effect of GC toward nitrocellulose based films, probably due to hydrogen bond interactions between GC and nitrocellulose. The GC efficiency gives the possibility to decrease the content of the plasticizer in the formulation.

GC can be thus considered as a biobased ingredient abiding by the green chemistry concepts, and safe enough to be used in an ecodesigned nail polish formulation.

## Data Availability

All datasets generated for this study are included in the manuscript and/or the supplementary files.

## Author Contributions

PdC wrote the evaluation of plasticizing properties and the results and discussion on this part. MB was the Ph.D. student who worked on this subject. CC wrote the introduction and conclusion and the “mini-review” part on synthesis of glycerol carbonate by biocatalysis and from crude glycerol. MU wrote the “mini-review” part on synthesis of glycerol carbonate by glycerolysis with urea and reaction from CO_2_. ST-R wrote the abstract, the mini-review part on synthesis of glycerol carbonate by transcarbonatation, and the results and discussion on the improvement of this route.

### Conflict of Interest Statement

The authors declare that the research was conducted in the absence of any commercial or financial relationships that could be construed as a potential conflict of interest.

## Footnotes

^1^Top Value Added Chemicals from Biomass Volume I–Results of Screening for Potential Candidates from Sugars and Synthesis Gas. Report August 2004. Available online at: https://www.nrel.gov/docs/fy04osti/35523.pdf (accessed February 28, 2019).

## References

[B1] Algoufi Y. T. Akpan U. G. Kabir G. Asif M. Hameed B. H. (2017). Upgrading of glycerol from biodiesel synthesis with dimethyl carbonate on reusable Sr-Al mixed oxide catalysts. Energy Conv. Manag. 138, 183–189. 10.1016/j.enconman.2017.01.078

[B2] Aresta M. Dibenedetto A. Nocito F. Ferragina C. (2009). Valorization of bio-glycerol: new catalytic materials for the synthesisof glycerol carbonateviaglycerolysis of urea. J. Catal. 268, 106–114. 10.1016/j.jcat.2009.09.008

[B3] Aresta M. Dibenedetto A. Nocito F. Pastore C. (2006). A study on the carboxylation of glycerol to glycerol carbonate with carbon dioxide: the role of the catalyst, solvent and reaction conditions. J. Mol. Catal. A Chem. 257, 149–153. 10.1016/j.molcata.2006.05.021

[B4] Averous L. Caillol S. Cramail H. (2017). Polymères biosourcés; perspectives et enjeux. Actualité Chimique, 422-423, 68–75.

[B5] Bai R. Wang S. Mei F. Li T. Li G. (2011). Synthesis of glycerol carbonate from glycerol and dimethyl carbonate catalyzed by KF modified hydroxyapatite. J. Ind. Eng. Chem. 17, 777–781. 10.1016/j.jiec.2011.05.027

[B6] Baker F. S. Jones M. Lewis T.J Privett G. Crofton D. J. Pethrick R. A. (1984). Dielectric studies of nitrocellulose-nitroglycerine mixtures. Polymer 25, 6, 815–820. 10.1016/0032-3861(84)90012-0

[B7] Bandres M. Deswartvaegher A. de Caro P. Senet J.-P. Thiebaud-Roux S. (2010). Plasticizer of Natural Origin for Nail Polish. U.S. Patent US 2010158835; French patent FR2895905, WO 2007080172. Bergerac: Chromadurlin.

[B8] Claude S. Mouloungui Z. Yoo J.-W. Gaset A. (2000). Method for Preparing Glycerol Carbonate. United state patent US 6025504. (Paris: Onidol).

[B9] Clements J. H. (2003). Reactive applications of cyclic alkylene carbonates. Ind. Eng. Chem. Res. 42, 663–674. 10.1021/ie020678i

[B10] Climent M. J. Corma A. Frutos P. Iborra S. Noy M. Velty A. (2010). Chemicals from biomass: synthesis of glycerol carbonate by transesterification and carbonylation with urea with hydrotalcite catalysts. The role of acid-base pairs. J. Catal. 269, 140–149. 10.1016/j.jcat.2009.11.001

[B11] Dabral S. Schaub T. (2019). The use of carbon dioxide (CO2) as a building block in organic synthesis from an industrial perspective. Adv. Syn. Catal. 361, 223–246. 10.1002/adsc.201801215

[B12] Du M. Li Q. Dong W. Geng T. Jiang Y. (2012). Synthesis of glycerol carbonate from glycerol and dimethyl carbonate catalyzed by K2CO3/MgO. Res. Chem. Intermed. 38, 1069–1077. 10.1007/s11164-011-0443-3

[B13] Du Y. Gao J. Kong W. Zhou L. Ma L. He Y. (2018). Enzymatic synthesis of glycerol carbonate using a lipase immobilized on magnetic organosilica nanoflowers as a catalyst. ACS Omega 3, 6642–6650. 10.1021/acsomega.8b00746 30023956 PMC6044822

[B14] Fernandes G. P. Yadav G. D. (2013). Selective glycerolysis of urea to glycerol carbonate using combustion synthesized magnesium oxide as catalyst. Catal. Today 309, 153–160. 10.1016/j.cattod.2017.08.021

[B15] Fujita S. I. Yamanishi Y. Arai M. (2013). Synthesis of glycerol carbonate from glycerol using zinc-containing solid catalysts: a homogeneous reaction. J. Catal. 297, 137–141. 10.1016/j.jcat.2012.10.001

[B16] George J. Patel Y Pillai M. S. Munshi P. (2009). Methanol assisted selective formation of 1,2-glycerol carbonate from glycerol and carbon dioxide using nBu2SnO as a catalyst. J. Mol. Catal. 304, 1–7. 10.1016/j.molcata.2009.01.010

[B17] Global Bio-Based Chemicals Market Forecast 2019–2027. Report February 2018. Available online at: https://www.reportlinker.com/p05001382/Global-Bio-Based-Chemicals-Market-Forecast.html (accessed February 28, 2019).,

[B18] Hérault D. Boutty B. Zander L. Strube A. (2003). Method for Producing Glycerol Carbonate. International patent WO 03022829. (Düsseldorf: Cognis Deutschland GmbH).

[B19] Hirotsu K. Kaneko T. (2001). Method for Producing 4-Hydromethyl-1,3-Dioxolan-2-One. Japanese patent JP2001172277. (Yamaguchi: Ube Industries Ltd.)

[B20] Hu S. Luo X. Wan C. Li Y. (2012). Characterization of crude glycerol from biodiesel plants. J. Agric. Food Chem. 60, 5915–5921. 10.1021/jf3008629 22612334

[B21] Indran V. P. Saud A. S. Maniam G. P. Yasoff M. M. Taufiq-Yap Y. H. Rahim M. H. A. (2016). Versatile boiler ash containing potassium silicate for the synthesis of organic carbonates. RSC Adv. 6, 34877–34884. 10.1039/C5RA26286K

[B22] Ishak Z. I. Sairi N. A. Alias Y. Aroua M. K. T. Yusoff R. (2016). Production of glycerol carbonate from glycerol with the aid of ionic liquid as catalyst. Chem. Eng. J. 297, 128–138. 10.1016/j.cej.2016.03.104

[B23] Ishak Z. I. Sairi N. A. Alias Y. Aroua M. K. T. Yusoff R. (2017). A review of ionic liquids as catalysts for transesterification reactions of biodiesel and glycerol carbonate production. Catal. Rev. 59, 44–49. 10.1080/01614940.2016.1268021

[B24] Jagadeeswaraiah K. Ch Ramesh K. Sai Prasad P. S. Loridant S. Lingaiah N. (2014). Synthesis of glycerol carbonate from glycerol and urea over tin-tungsten mixed oxide catalysts. App.Catal. A Gen. 469, 165–172. 10.1016/j.apcata.2013.09.041

[B25] Jung H. Lee Y. Kim D. Han S. Kim O. S. W. Lee J. (2012). Enzymatic production of glycerol carbonate from by-product Park. Enzyme Microb. Technol. 51, 143–147. 10.1016/j.enzmictec.2012.05.004 22759533

[B26] Kim K. H. Lee E. Y. (2018). Simultaneous production of transformer insulating oil and value-added glycerol carbonates from soybean oil by lipase-catalyzed transesterification in dimethyl carbonate. Energies 11:82 10.3390/en11010082

[B27] Kong P. S. Aroua M. K. Wan Daud W. M. A. (2016). Conversion of crude and pure glycerol into derivatives: a feasibility evaluation. Renew. Sust. Energy Rev. 63, 533–555. 10.1016/j.rser.2016.05.054

[B28] Lanjekar K. Rathod V. K. (2013). Use of glycerol for the production of glycerol carbonates through the greener route. J. Environ. Chem. Eng. 1, 1231–1236. 10.1016/j.jece.2013.09.015

[B29] Li H. Jiao X. Li L. Zhao N. Xiao F. Wei W. (2015). Synthesis of glycerol carbonate by direct carbonylation of glycerol with CO2 over solid catalysts derived from Zn/Al/La and Zn/Al/La/M(M = Li, Mg and Zr) hydrotalcites. Catal. Sci. Technol. 5, 989–1005. 10.1039/C4CY01237B

[B30] Li J. Wang T. (2010). Coupling reaction and azeotropic distillation for the synthesis of glycerol carbonate from glycerol and dimethyl carbonate. Chem. Eng. Proc. 49, 530–535. 10.1016/j.cep.2010.04.003

[B31] Li J. Wang T. (2011). Chemical equilibrium of glycerol carbonate synthesis from glycerol. J. Chem. Thermodyn. 43, 731–736. 10.1016/j.jct.2010.12.013

[B32] Li W. Sreerangappa R. Estager J. Monbaliu J.-C. Debecker D. P. Luis P. (2018). Application of perevaporation in the bio-production of glycerol carbonate. Chem. Eng. Proc. Intensif. 132, 127–136. 10.1016/j.cep.2018.08.014

[B33] Li Y. Liu J. He D. (2018). Catalytic synthesis of glycerol carbonate from biomass-based glycerol and dimethyl carbonate over Li-La2O3. Appl. Catal. A Gen. 564, 234–242. 10.1016/j.apcata.2018.07.032

[B34] Liu J. Li Y. Liu H. He D. (2018). Transformation of CO2 and glycerol to glycerol carbonate over CeO2/ZrO2 solid solution-effect of Zr doping. Biomass Bioenergy 118, 74–83. 10.1016/j.biombioe.2018.08.004

[B35] Liu J. Li Y. Zhang J. He D. (2016). Glycerol carbonylation with CO2 to glycerol carbonate over CeO2 catalyst and the influence of CeO2 preparation methods and reaction parameters. Appl. Catal. A Gen. 513, 9–18. 10.1016/j.apcata.2015.12.030

[B36] Liu Z. Wang J. Kang M. Yin N. Wang X. Tan Y. (2015). Structure activity correlations of LiNO3/ Mg4AlO5.5 catalysts for glycerol carbonate synthesis from glycerol and dimethyl carbonate. J. Ind. Eng. Chem. 21, 394–399. 10.1016/j.jiec.2014.02.051

[B37] Lu P. Wang H. Hu K. (2013). Synthesis of glycerol carbonate from glycerol and dimethyl carbonate over the extruded CaO-based catalysts. Chem. Eng. J. 228, 147–154. 10.1016/j.cej.2013.04.109

[B38] Luo X. Ge X. Cui S. Li Y. (2016). Value-added processing of crude glycerol into chemicals and polymers. Biores. Technol. 215, 144–154. 10.1016/j.biortech.2016.03.042 27004448

[B39] Ma J. Song J. Liu H. Liu J. Zhang Z. Jiang T. (2012). One-pot conversion of CO2 and glycerol to value-added products using propylene oxide as the coupling agent. Green Chem. 14, 1743–1748. 10.1039/c2gc35150a

[B40] Munshi M. K. Biradar P. S. Gade S. M. Rane V. H. Kelkar A. A. (2014a). Efficient synthesis of glycerol carbonate/glycidol using 1,8-diazabicyclo[5.4.0]undec-7-ene (DBU) based ionic liquids as catalyst. RSC Adv. 4, 17124–17128. 10.1039/c3ra47433j

[B41] Munshi M. K. Gade S. M. Mane V. M. Mishra D. Pal S. Vanka K. (2014b). 1,8-diazabicyclo[5.4.0]undec-7-ene (DBU): a highly efficient catalyst in glycerol carbonate synthesis. J. Mol.Catal. A Chem. 391, 144–149. 10.1016/j.molcata.2014.04.016

[B42] Naik U. Petitjean L. Refes K. Picquet M. Plasseraud L. (2009). Imidazolium-2-carboxylate as an efficient, expeditious and eco-friendly organocatalyst for glycerol carbonate synthesis. Adv. Synth. Catal. 351, 1753–1756. 10.1002/adsc.200900280

[B43] Nanda M. R. Yuan Z. Qin W. Poirier M. A. Chunbao X. (2014). Purification of crude glycerol using acidification: effects of acid types and product characterization. Austin J. Chem. Eng. 1, 1–7. Available online at: https://scholar.google.com/scholar_lookup?title=Purification%20of%20crude%20glycerol%20using%20acidification%3A%20effects%20of%20acid%20types%20and%20product%20characterization&publication_year=2014&author=M.%20Nanda&author=Z.%20Yuan&author=W.%20Qin.

[B44] Nguyen N. Demirel Y. (2013). Economic analysis of biodiesel and glycerol carbonate production plant by glycerolysis. J. Sust. Bioenergy Syst. 3, 209–216. 10.4236/jsbs.2013.33029

[B45] Nguyen-Phu H. Park C. Woo Shin E. (2018). Dual catalysis over ZnAl mixed oxides in the glycerolysis of urea: homogeneous and heterogeneous reaction routes. Appl. Catal. A Gen. 552, 1–10. 10.1016/j.apcata.2017.12.018

[B46] Ochoa-Gómez J. R. Gómez-Jiménez-Aberasturi O. Ramirez-López C. Belsué M. (2012). A brief review on industrial alternatives for manufacturing of glycerol carbonate, a green chemical. Org. Process Res. Dev. 16, 389–399. 10.1021/op200369v

[B47] Okoye P. U. Hameed B. H. (2016). Review on recent progress in catalytic carboxylation and acetylation of glycerol as a byproduct of biodiesel production. Renew. Sust. Energy Rev. 53, 558–574. 10.1016/j.rser.2015.08.064

[B48] Paroo Indran V. Sajidah Haji Saud A. Pragas Maniam G. Taufiq-Yap Y Hasbi Ab Rahim M. (2017). Viable glycerol carbonate synthesis through direct crude glycerol utilization from biodiesel industry. Waste Biomass Valor. 8, 1049–1059. 10.1007/s12649-016-9681-3

[B49] Paroo Indran V. Syuhada Zuhaimi N. Asyrak Deraman M. Pragas Maniam G. Mohd Yusoff M. Yun Hin T. (2014). An accelerated route of glycerol carbonate formation from glycerol using waste boiler ash as catalyst. RSC Adv. 4, 25257–25267. 10.1039/C4RA02910K

[B50] Rokicki G. Rakoczy P. Parzuchowski P. Sobiecki M. (2005). Hyperbranched aliphatic polyethers obtained from environmentally benign monomer: glycerol carbonate. Green Chem. 7, 529–539. 10.1039/b501597a

[B51] Roschat W. Phewphong S. Kaewpuang T. Promarak V. (2018). Synthesis of glycerol carbonate from transesterication of glycerol with dimethyl carbonate catalyzed by CaO from natural sources as green and economical catalyst. Mater. Today Proc. 5, 3909–3915. 10.1016/j.matpr.2018.02.039

[B52] Song Q. W. Zhou Z. H. He L. N. (2017). Efficient, selective and sustainable catalysis of carbon dioxide. Green Chem. 19, 3707–3728. 10.1039/C7GC00199A

[B53] Song X. Wu Y. Cai F. Pan D. Xiao G. (2017). High efficiency and low-cost Li/ZnO catalysts for synthesis of glycerol carbonate from glycerol transesterification: the role of Li and ZnO interaction. Appl.Catal. A Gen. 532, 77–85. 10.1016/j.apcata.2016.12.019

[B54] Song X. Wu Y. Pan D. Zhang J. Xu S. Gao L. (2018).Functionalized DVB-based polymer catalysts for glycerol and CO2 catalytic conversion. J. CO2 Utiliz. 28, 326–334. 10.1016/j.jcou.2018.10.015

[B55] Sonnati M. O. Amigoni S. Taffin de Givenchy E. P. Darmanin T. Choulet O. Guittard F. (2013). Glycerol carbonate as a versatile building block for tomorrow: synthesis, reactivity, properties and applications. Green Chem. 15, 283–306. 10.1039/C2GC36525A

[B56] Teng W. K. Ngoh G. C. Yusoff R. Aroua M. K. (2014).A review on the performance of glycerol carbonate production via catalytic transesterification: effects of influencing parameters. Energy Convers. Manag. 88, 484–497. 10.1016/j.enconman.2014.08.036

[B57] Teng W. K. Ngoh G. C. Yusoff R. Aroua M. K. (2016). Microwave-assisted transesterification of industrial grade crude glycerol for production of glycerol carbonate. Chem. Eng. J. 284, 469–477. 10.1016/j.cej.2015.08.108

[B58] Tudorache M. Negoi A. Tudora B. Parvulescu V. I. (2014). Environmental-friendly strategy for biocatalytic conversion of waste glycerol to glycerol carbonate. Appl. Catal. B Env. 146, 274–278. 10.1016/j.apcatb.2013.02.049

[B59] Van Mileghem S. De Borggraeve W. M. Baxendale I. R. (2018). A robust and scalable continuous flow process for glycerol carbonate, demonstrating that GC could be produced continuously at a pilot scale although the industrial processes currently used are batch ones. Chem. Eng. Technol. 41, 2014–2023. 10.1002/ceat.201800012

[B60] Wan D. Zhang X. Cong X. Liu S. Zhou D. (2018). Influence of Zr on the performance of Mg-Al catalysts via hydrotalcite-like precursors for the synthesis of glycerol carbonate from urea and glycerol. Appl. Catal. A Gen. 555, 36–46. 10.1016/j.apcata.2018.02.009

[B61] Wan Y. Lei Y. Lan G. Liu D. Li G. Bai R. (2018). Synthesis of glycerol carbonate from glycerol and dimethyl carbonate over DABCO embedded porous organic polymer as a bifunctional and robust catalyst. Appl. Catal. A Gen. 562, 267–275. 10.1016/j.apcata.2018.06.022

[B62] Wang S. Hao P. Li S. Zhang A. Guan Y. Zhang L. (2017). Synthesis of glycerol carbonate from glycerol and dimethyl carbonate catalyzed by calcined silicates. Appl. Catal. A Gen. 542, 174–181. 10.1016/j.apcata.2017.05.021

[B63] Wu Y. Song X. Cai F. Xiao G. (2017). Synthesis of glycerol carbonate from glycerol and diethyl carbonate over Ce-NiO catalyst: the role of multiphase Ni. J. Alloys Comp. 720, 360–368. 10.1016/j.jallcom.2017.05.292

[B64] Wu Y. Song X. Zhang J. Li S. Yang X. Wang H. (2018). Synthesis of glycerol carbonate from glycerol and diethyl carbonate over CeO2-CdO catalyst: the role of Ce4+ doped into CdO lattice. J. Taïwan Inst. Chem. Eng. 87, 131–139. 10.1016/j.jtice.2018.03.023

[B65] Wypych G. (2017). Handbook of Plasticizers, 3rd edn. Toronto, ON: ChemTec Publishing 10.1016/B978-1-895198-96-6.50005-5

[B66] Yuan Y. A. (2015). Effect of glycerol carbonate as plasticizer on P(AN-MMA) polymer electrolyte. Appl. Mech. Mater. 713–715, 2658–2662. 10.4028/www.scientific.net/AMM.713-715.2658

[B67] Zhang P. Liu L. Fan M. Dong Y. Jiang P. (2016). The value-added utilization of glycerol for the synthesis of glycerol carbonate catalyzed with a novel porous ZnO catalyst. RSC Adv. 6, 76223–76230. 10.1039/C6RA14288E

[B68] Zheng I. Xia S. Lu X. Hou Z. (2015). Transesterification of glycerol with dimethyl carbonate over calcined Ca-Al hydrocalumite. Chin. J. Catal. 36, 1759–1765. 10.1016/S1872-2067(15)60915-9

